# Effects of Decompression Treatment for Controlling the Powderpost Beetle, *Lyctus africanus* Lesne, (Coleoptera: Lyctinae)

**DOI:** 10.3390/insects7030036

**Published:** 2016-07-15

**Authors:** Kazushi Nakai, Tatsuya Hiraku, Izumi Fujimoto, Tsuyoshi Yoshimura

**Affiliations:** 1 Production Engineering and Procurement Department, Yamaha Corporation, 283 Aoya-cho, Minami-ku, Hamamatsu 435-8567, Japan; tatsuya.hiraku@music.yamaha.com; 2 Research Institute for Sustainable Humanosphere, Kyoto University, Uji Campus, Kyoto 611-0011, Japan; izumi_fujimoto@rish.kyoto-u.ac.jp (I.F.); tsuyoshi@rish.kyoto-u.ac.jp (T.Y.)

**Keywords:** decompression treatment, *L. africanus*, powderpost beetle, CO_2_ purge

## Abstract

The efficacy of decompression treatment as a non-destructive method to control larvae of the powderpost beetle, *Lyctus africanus* Lesne, was evaluated in the laboratory using various combinations of two pressure levels, 1.1 kPa and 40 kPa, and three temperature levels, 20, 25, and 40 °C. Larval mortality generally depended on weight reduction while decreases in the oxygen level had relatively little effect. The lower pressure, 1.1 kPa, significantly affected mortality, and no larvae survived after 12 h of this pressure treatment, at 25 °C. The average body weight was reduced with treatment time and temperature, and the reduction rate at 25 °C was higher than that at the lower temperature, 20 °C. Effects on larvae of the higher pressure treatment, 40 kPa, with a CO_2_ gas purge, were tested to determine the feasibility of decompression treatment in the manufacturing process. Although higher pressure resulted in low mortality, the body weight was dramatically decreased using the CO_2_ purge. These results present important information on the possibility of using decompression treatment for wood products.

## 1. Introduction

Both chemical and non-chemical treatments have been used to control pest insects in the manufacturing process for wood products such as musical instruments. Since many wood materials such as timber, laminated wood and plywood are processed and assembled to produce wood products, managing the pests in such raw materials is important for quality assurance in the final products.

Due to increasing public concern about environmental safety [1,2], non-chemical pest management has been extensively studied. Such methods have been used for pest management in museums, libraries and historic buildings because they convey a lower risk to materials and operators as well as the environment [3]. Inert gases such as carbon dioxide (CO_2_) gas are often used in non-chemical/non-destructive methods due to the low risk of damage to materials. In order to utilize such treatments efficiently, the specific killing effect of these gases to all stages of the target insects living inside the wood material must be clarified. Kigawa et al. (2011) tested the effects of CO_2_ gas on some pest insects comparing it with the fumigation treatment by sulfuryl fluoride, and found that the mortality in painted timbers of the larvae of two species, *Priobium cylindricum* and *Sitophilus zeamais*, did not reach 100% [4]. Applications of heat treatment including microwave irradiation have also been tested as alternatives [5,6,7,8,9,10,11,12,13,14,15,16]. In particular, short-term microwave exposure has been shown to produce higher mortality than topical insecticide applications and is comparable to fumigation while being less damaging than high-temperature treatments [5,6,7,8,9,10,11,12,13,14,15,16].

Lyctinae beetles are called “powderpost beetles” due to the way they expel wood frass when they emerge from infested wood materials. *Lyctus brunneus* (Stephens) has been studied extensively as one of the most important species of drywood pest insects [17,18]. The beetle larvae live inside the wood materials, spending several months to one year before emerging after becoming adults [19]. Therefore, the Lyctinae beetles in the materials are difficult to detect in early stages of infestation. *Lyctus africanus* Lesne is also an important species having very similar anatomical and ecological features to those of *L. brunneus* [18,19,20]. The two species have similar thermal tolerance [21]. In Japan, infestations of *L. africanus* have even become more common than those of *L. brunneus*, especially in the southern part of the main islands [22]; thus, *L. africanus* must be considered a key target when thinking about the development of a country-wide management system.

In general, the manufacturing of wood products such as musical instruments is comprised of four basic processes: drying, processing, painting, and assembling including adhesion. Wood products for musical instruments must maintain low moisture content to ensure the quality of the products, and it is well known that larvae of Lyctinae beetles need low-moisture-content wood for their survival, optimally conditioned at 16%. The final target of the present study was to develop a novel insect management system adapted to the manufacturing process of wood musical instruments. The essential requirements we sought to fulfill were rapid treatment and high mortality without harming products or the environment.

For this purpose, fundamental data on decompression treatments applicable for the economically important powderpost beetle, *L. africanus*, are discussed together with the results regarding the effects of low-pressure treatment on another economically important species, *L. brunneus*.

## 2. Materials and Methods

### 2.1. Insects

This study utilized *L. africanus* larvae over 2 mm in length obtained from a laboratory colony maintained in the Deterioration Organisms Laboratory (DOL), Research Institute for Sustainable Humanosphere at Kyoto University for more than 20 years in a dark environmental chamber at 26 °C and 65% relative humidity (RH) [23]. For *L. brunneus*, larvae over 3 mm in length were also prepared from a laboratory colony which has been maintained in similar conditions to those of *L. africanus*. 

### 2.2. Weight Reduction Rate of Larvae

The moisture contents of larvae of *L. africanus* were measured by kiln drying in order to obtain base data for the evaluation of moisture evaporation from the body. Closed Petri dishes with 10 larvae were dried in temperature-controlled chambers at four different temperatures, 25, 40, 60 and 100 °C. The change of weight was monitored after 1, 3, 5, 7 and 22 h, and the weight reduction during a given time was calculated. The weight reduction rate, *W_R_*, was calculated by the following Equation (1):

(1) $$W_{R}\;=\;\frac{W_{0}-\;W_{1}}{W_{0}}\;\times \;100\;\left(\%\right)$$

where *W_0_* is the total weight of 10 larvae before treatment, and *W_1_* is the total weight of 10 larvae after treatment. *W_1_* was measured 30 min afterincubation at room temperature and 45% ± 5% RH.

### 2.3. Experimental Apparatus and Decompression Treatment

#### 2.3.1. Experimental Apparatus and Conditions

**Figure 1:**
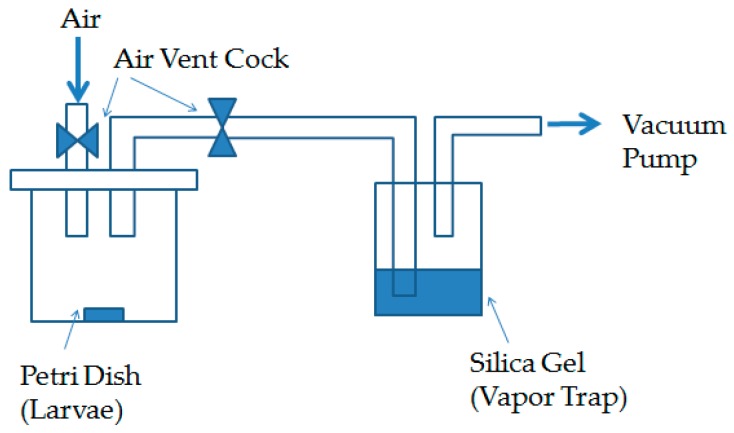
Experimental set-up for the high vacuum treatment (1.1 kPa).

A cylindrical acrylic chamber (320 mm diamater, 500 mm height) was used for decompression treatment. A closed glass Petri dish (75 mm diameter, 20 mm height) with 10 larvae was placed on the bottom of the chamber (Figure 1). In the decompression process, the pressure of the chamber was decreased by a vacuum pump (BSEW-150, Sato Vac Inc, Tokyo, Japan) at 20–25 °C and 45% ± 5% RH. In this study, two levels of pressure, 1.1 kPa or 40 kPa, were used. For the sake of convenience, 1.1 kPa was regarded as the high vacuum condition (lower pressure), and 40 kPa was defined as the low vacuum condition (higher pressure). The high vacuum condition (1.1 kPa) was designed to determine the lethal conditions for the larvae of *L. africanus*, and the low vacuum condition was used to evaluate the feasibility of the decompression treatment in the manufacturing process.

#### 2.3.2. Treatment in the High Vacuum Condition

The pressure inside the acrylic chamber holding the test insects was kept at 1.1 kPa for 16 h at three different temperatures, 20, 25 and 40 °C, in a temperature-controlled room. After the test period, the pressure was rapidly normalized with atmospheric pressure (approximately 100 kPa). In addition, other closed Petri dishes with test insects were kept in temperature-controlled chambers at three different temperatures, 20, 25 and 40 °C, under normal pressure as control samples.

#### 2.3.3. Treatment in the Low Vacuum Condition

**Figure 2:**
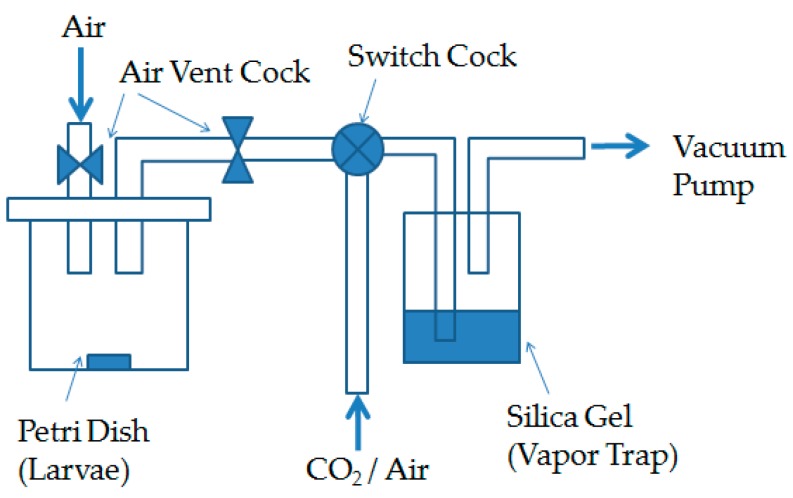
Experimental set-up for the low vacuum treatment under CO_2_ or air (40 kPa).

Figure 2 shows the experimental set-up for the low vacuum treatment. A CO_2_ bottle was connected to the acrylic chamber as with the high vacuum treatment in Figure 1. Two atmospheric conditions were used in this experiment: in the first, the chamber was vacuumed to 1.1 kPa, and then purged to 40 kPa by CO_2_ (low vaccum + CO_2_); in the second, an air purge was substituted for CO_2_ (low vacuum + normal air). The chambers were then placed in a room at either 25 °C or 40 °C for 48 h. After the test period, the pressure was rapidly reduced to normal pressure as with the high vacuum treatment.

#### 2.3.4. Evaluation of Effects of Decompression Treatments on *L. africanus*

Effects of the decompression treatment were evaluated by both mortality and weight reduction rate, *W_R_*, of larvae.

Mortality was calculated by measuring the number of surviving larvae after 16 h of post-incubation at 26 °C, 65% RH. *W_R_* was also calculated by Equation (1).

### 2.4. Comparison of Effects of the High Vacuum Treatment on the Mortality of L. brunneus

The larvae of the two species, *L. africanus* and *L. brunneus*, were prepared to compare the effects of the high vacuum treatment. Closed glass Petri dishes (75 mm diameter, 20 mm height) with 10 larvae were set-up with the same procedure as described in Section 2.3.1. They were exposed to a high vacuum condition (1.1 kPa) (Figure 1), and kept up to 4 h at 40 °C in a temperature-controlled room. Pressure was rapidly returned to normal (approximately 100 kPa) after exposure.

Mortality and *W_R_* of larvae were calculated based on the procedure shown in Section 2.3.4.

### 2.5. Statistical Analysis

Both average mortality and *W_R_* were compared respectively by one-way analysis of variance (ANOVA) to analyze the effect of each treatment condition. The Tukey-Kramer test at 5% critical difference was also used as a supplementary test.

## 3. Results and Discussion

### 3.1. Moisture Reduction of Larvae by Heat Treatment

**Figure 3:**
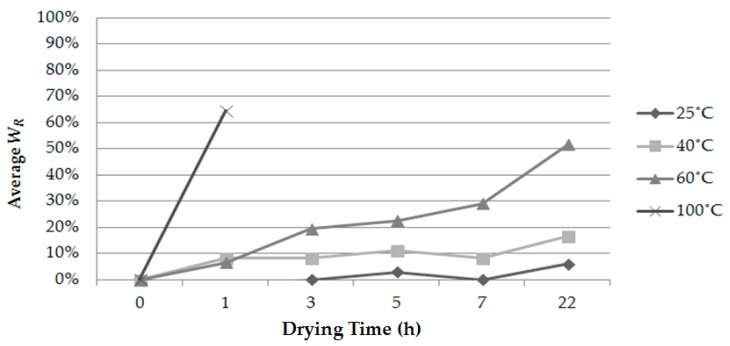
Average weight reduction rates of larvae of *L. africanus* after drying at 25, 40, 60 and 100 °C for 1, 3, 5, 7 and 22 h (*n* = 3).

Figure 3 shows the weight reduction rates of larvae of *L. africanus* at 25, 40, 60 and 100 °C for 1, 3, 5, 7 and 22 h. The weight of larvae dramatically decreased to about 35% of the original weight after 1 h with complete mortality at 100 °C. The shape of exposed larvae was significantly different from that of the unexposed larvae, and the color of skin became brownish.

In contrast, the weight reduction rates at both 40 °C and 60 °C for 1 h were only about 10%. Treatment for over 5 h at 60 °C affected larvae mortality, and no larvae exposed for over 7 h could survive due to their >30% body weight loss. Neither the 25 °C nor 40 °C heat treatments affected the survival of larvae, and their weights varied less during the drying time. As a result, it was estimated that heat treatment below 40 °C for 22 h under normal air pressure had no detrimental effect on the larvae of *L. africanus.*

### 3.2. Lethal Condition at High Vacuum Treatment

**Figure 4:**
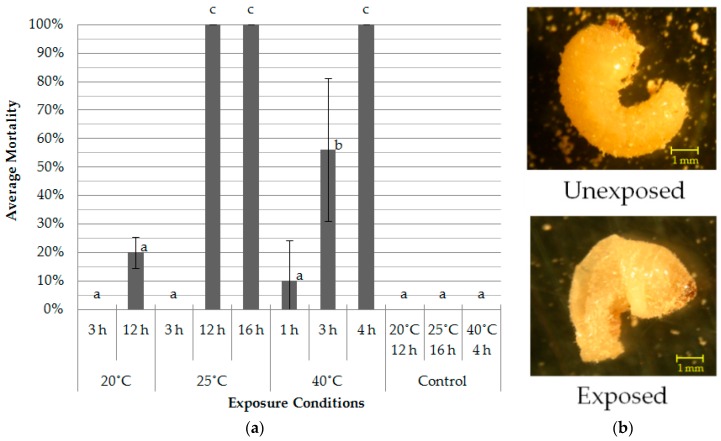
Mortality and the change of shape of *L. africanus* larvae by the high vacuum treatment (*n* = 5). (**a**) Mortality (mean ± SD) after decompression treatment at 1.1 kPa, (**b**) Photographs of larvae of *L. africanus* before (upper) and after treatment (bottom) at 25 °C, 1.1 kPa for 12 h. For each condition, means with the same letters are not significantly different (Tukey-Kramer test; *p* < 0.05) following one-way analysis of variance (ANOVA).

Figure 4a shows average moltalities of the larvae after the decompression treatment at 1.1 kPa under three different temperatures. All the larvae died after 12-h and 16-h treatments at 25 °C. The larvae showed completely different shapes after treatment due to drastic water content reduction (Figure 4b). The body volume of the larvae increased when pressure was decreased to 1.1 kPa. Such volume increase was maintained during the treatment, and it decreased simultaneously with the increase of pressure after treatment. This result may suggest that a decompressed environment could remove air from the body water and promote vaporization.

Mortality under 40 °C treatment significantly increased for the first 4 h. In contrast, the treatment at 20 °C for 12 h resulted in only about 20% mortality, while the 25 °C treatment showed 100% mortality after 12 h. This phenomenon was probably due to the relationship between temperature and water vapour pressure; the longer exposure time will achieve higher mortality even at the 20 °C treatment. Furthermore, temperature apparently also contributed to the high mortality of insects. As a result, more than 12 h of the 25 °C treatment or 4 h of the 40 °C treatment were considered to be lethal conditions for *L. africanus* larvae.

Table 1 shows the average weight reduction rates (*W_R_*) for 5 treatment conditions. The weight of larvae was decreased to approximately 50% of the original weight at one of the lethal conditions, 25 °C for 12 h (Figure 4a, Table 1). Such reduction is equal to about 80% of their original water content, which was measured at about 65% by kiln drying at 100 °C for 1 h (Figure 3). Interestingly, higher mortality was obtained after 4 h of the 40 °C treatment, although *W_R_* was almost equal to that of the 12-h 20 °C treatment, which showed no effect on mortality (Figure 4a). This probably is a result of the higher temperature as well as low pressure.

Although the decrease in oxygen level was apparent under this pressure condition, such an effect is probably not a major reason for high mortality. Navarro and Calderon (1980) in their testing methods on three stored product insects (adults), reported that the exposure time became shorter with increased temperature, and the reduced treatment time may be due to a rise in temperature [20]. Therefore, the heating effect may be the major factor in killing the insects, and the 40 °C treatment is likely to have a serious effect on larvae mortality while dehydration can affect mortality at lower temperature.

### 3.3. Effects of Low Vacuum Treatment

**Figure 5:**
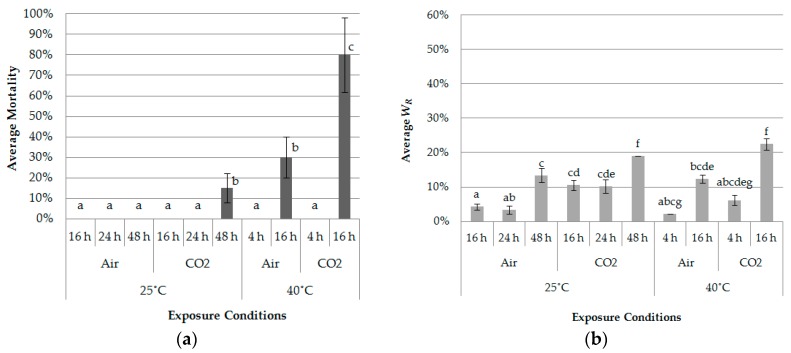
Average mortality and *W_R_* of larvae of *L. africanus* after the low vacuum treatment at 20–25 °C and 45% ± 5% relative humidity (RH) with air purge and CO_2_ purge (*n* = 5): (**a**) Average mortality (mean ± SD) after low vacuum treatments (40 kPa) and purging; (**b**) Average *W_R_* (mean ± SD) after the low vacuum treatments (40 kPa) and purging. For each condition, means followed by the same letter are not significantly different (Tukey-Kramer test; *p* < 0.05) following one-way ANOVA.

Figure 5a,b show average mortality and *W_R_* of the larvae after exposure under 40 kPa for 11 combinations of duration and temperature. As expected, both parameters were lower than those in the high vacuum treatments due to the relationship between air pressure and water vapour pressure. In general, the rate of water evaporation speeds up under the lower pressure conditions compared to normal pressure, because the environmental pressure becomes lower than the water vapour pressure. Therefore, the 40 kPa treatment might be regarded as the unfavorable condition regarding the drying effect of decompression treatment.

The average mortality among air-purged and CO_2_-purged treatments was significantly different (25 °C for 48-h: *F* = 16.00, df = 1, *p* = 0.0039; 40 °C for 16-h: *F* = 77.93, df = 1, *p* = 0.00031) (Figure 5a). Treatments at 40 °C caused higher mortality than those at 25 °C, under the high vacuum condition (1.1 kPa). In addition, the use of CO_2_ as a purging gas resulted in higher mortality and *W_R_* at both 25 °C and 40 °C. As shown in Figure 5b, *W_R_* values with CO_2_ purging were 2-fold greater compared to air purging. This may depend on the difference of relative humidity between containers in which the two gases were purged. The relative humidity in each chamber was controlled for air and CO_2_ purging. In this study, the original air was completely released in preparation for the high vacuum condition (1.1 kPa). Each gas was purged after 1.1 kPa, obviously resulting in the different levels of relative humidity in each chamber. Air purging was introduced under laboratory room conditions (20–25 °C, 45% ± 5% RH) in the chamber, while CO_2_ purging did not bring any moisture into the chamber. Finally, the relative humidity for CO_2_ purging was kept lower than that for air purging. The rate of water evaporation can increase at low relative humidity; thus, CO_2_ purging probably has an advantage for leading to higher mortality in the drying process. The role of relative humidity in some insects was studied by several reseachers, and it is suggested that lower humidity promoted the death of insects at the same gas composition [24,25].

CO_2_ gas itself can directly affect mortality, even if *W_R_* is lower than the lethal reduction rate. The effect of low oxygen or high CO_2_ concentrations on some insects have been previously investigated [4,24,25]. Banks (1978) suggested that oxygen concentrations below about 2% were needed for high mortality in stored product insects [26]. This suggests that the oxygen concentration might be an important factor for eradicating beetles in addition to the drying effect caused by a low pressure environment.

### 3.4. Comparison of Effects of the High Vaccum Treatment against L. africanus and L. brunneus

Table 2 shows average mortality and *W_R_* fin larvae of *L. africanus* and *L. brunneus* after high vacuum (1.1 kPa) treatment at 40 °C for 4 h. However, there were no significant differences in mortality or *W_R_* (*F* = 1.30, df = 1, *p* = 0.38) between the two Lyctinae species. These two species are morphologically quite similar [20]. From these results, it is possible that *L. africanus* and *L. brunneus* react similarly to environmental conditions close to the lethal level.

Mortality of an insect depends on the tolerance of such external factors as thermal shock or air composition. Differences in lethal temperature for some closely related species have been studied [5,10,21], and *L. africanus* was reported to have similar thermal tolerance to that of *L. brunneus* [21]. Rust and Kennedy (1993) suggested that *Lyctus* spp. such as *L. brunneus* and *L. linearis* were similarly affected by environmental conditions [27]. According to such studies and the present results, it may be possible to apply the same lethal condition (temperature, pressure, and time) to all Lyctinae species.

## 4. Conclusions 

In this study, the lethal conditions of decompression treatment with a specified pressure for larvae of *L. africanus* were analyzed, and the killing mechanism was also discussed. Decompression treatment with less than 40 kPa could cause high mortality of larvae of Lyctus beetles. The lower pressure (1.1 kPa) achieved complete mortality after 12 h at 25 °C and 4 h at 40 °C, and the higher pressure (40 kPa) with CO_2_ gas purging also resulted in high mortality after 16 h at 40 °C. *W_R_* values for higher pressure with CO_2_ was estimated to be about twice that without CO_2_, and suggests that a dehydration effect on larvae was achieved with such a process.

Further research will be needed that tests the effectiveness and practicality of decompression treatments of larvae, and other live stages of the beetles such as eggs, adults and pupae in large and/or painted wood materials.

## Figures and Tables

**Table 1:** Average weight reduction rates (*W_R_*) (% (mean ± SD)) for the 5 treatments (*n* = 5).

Treatment Condition		*W_R_* *
Temperature (°C)	Time (h)	
20	12	40.74 ± 6.13 ^a^
25	12	48.71 ± 3.16 ^a^
40	1	1.31 ± 6.01 ^b^
40	3	15.80 ± 16.20 ^bc^
40	4	35.24 ± 1.07 ^ac^

* Means with the same letter are not significantly different (Tukey-Kramer test; *p* < 0.05) following one-way ANOVA.

**Table 2:** Average mortality and *W_R_* of larvae of *L. africunus* and *L. brunneus* (% (mean ± SD)) after treatment at 40 °C and 1.1 kPa for 4 h (*n* = 3).

Species	Mortality	*W_R_* *
*Lyctus africanus*	100 ± 0	35.24 ± 1.07 ^a^
*Lyctus brunneus*	100 ± 0	36.17 ± 0.42 ^a^

* Means followed by the same letter were not significantly different (Tukey-Kramer test; *p* < 0.05) following one-way ANOVA.
